# Deep-Learning-Based Automatic Segmentation of Head and Neck Organs for Radiation Therapy in Dogs

**DOI:** 10.3389/fvets.2021.721612

**Published:** 2021-09-06

**Authors:** Jeongsu Park, Byoungsu Choi, Jaeeun Ko, Jaehee Chun, Inkyung Park, Juyoung Lee, Jayon Kim, Jaehwan Kim, Kidong Eom, Jin Sung Kim

**Affiliations:** ^1^Department of Veterinary Medical Imaging, College of Veterinary Medicine, Konkuk University, Seoul, South Korea; ^2^Department of Radiation Oncology, Yonsei Cancer Center, Yonsei University College of Medicine, Seoul, South Korea; ^3^Department of Integrative Medicine, Yonsei Cancer Center, Yonsei University College of Medicine, Seoul, South Korea

**Keywords:** radiation therapy, deep-learning-based automatic segmentation, head and neck cancer, dog head and neck, artificial intelligence

## Abstract

**Purpose:** This study was conducted to develop a deep learning-based automatic segmentation (DLBAS) model of head and neck organs for radiotherapy (RT) in dogs, and to evaluate the feasibility for delineating the RT planning.

**Materials and Methods:** The segmentation indicated that there were potentially 15 organs at risk (OARs) in the head and neck of dogs. Post-contrast computed tomography (CT) was performed in 90 dogs. The training and validation sets comprised 80 CT data sets, including 20 test sets. The accuracy of the segmentation was assessed using both the Dice similarity coefficient (DSC) and the Hausdorff distance (HD), and by referencing the expert contours as the ground truth. An additional 10 clinical test sets with relatively large displacement or deformation of organs were selected for verification in cancer patients. To evaluate the applicability in cancer patients, and the impact of expert intervention, three methods–HA, DLBAS, and the readjustment of the predicted data obtained via the DLBAS of the clinical test sets (HA_DLBAS)–were compared.

**Results:** The DLBAS model (in the 20 test sets) showed reliable DSC and HD values; it also had a short contouring time of ~3 s. The average (mean ± standard deviation) DSC (0.83 ± 0.04) and HD (2.71 ± 1.01 mm) values were similar to those of previous human studies. The DLBAS was highly accurate and had no large displacement of head and neck organs. However, the DLBAS in the 10 clinical test sets showed lower DSC (0.78 ± 0.11) and higher HD (4.30 ± 3.69 mm) values than those of the test sets. The HA_DLBAS was comparable to both the HA (DSC: 0.85 ± 0.06 and HD: 2.74 ± 1.18 mm) and DLBAS presented better comparison metrics and decreased statistical deviations (DSC: 0.94 ± 0.03 and HD: 2.30 ± 0.41 mm). In addition, the contouring time of HA_DLBAS (30 min) was less than that of HA (80 min).

**Conclusion:** In conclusion, HA_DLBAS method and the proposed DLBAS was highly consistent and robust in its performance. Thus, DLBAS has great potential as a single or supportive tool to the key process in RT planning.

## Introduction

Radiation therapy (RT) is one of the methods for cancer treatment that utilizes beams of intense energy to eliminate cancer cells. The use of RT in clinical practice has evolved over a long period (1). Veterinary facilities are both small in size and number when compared to that of human medicine facilities. Nevertheless, the clinical utilization of RT has increased in recent decades (2, 3).

Several procedures are used in RT, and organ segmentation is a prerequisite for quantitative analysis and RT planning (4). Organ segmentation is achieved by delineating along the boundaries of the organs at risk (OARs) and clinical target volumes (CTVs). The delineating process is commonly referred to as contouring (5). Currently, segmentations are manually achieved by experts during RT planning, especially, the three-dimensional conformal and intensity-modulated RT, as they require more accurate delineation of the CTVs and OARs (3, 6). However, delineation is challenging and time-consuming owing to the complexity of the structures involved. Moreover, this procedure requires considerable attention to detail and expertise in anatomy and imaging modality. Thus, this limits the sample size that can be analyzed properly (3, 6, 7). Furthermore, the outcome strongly depends on the skill of the observer, and hence a significant amount of inter-observer variation exists (8). A previous study showed that the contours from multiple observers overlapped with up to 60% of volume variations that could lead to substantial variations in RT planning (9). Practitioners in human medicine have overcome these limitations by using auto-segmentation techniques, which have gained significant attention for their potential use in routine clinical workflows (3). The current main research focus of RT is deep-learning-based auto-segmentation (DLBAS); this is the most recent method for automatic segmentation (3, 10–21).

In this study, DLBAS was conducted on the head and neck of dogs and subsequently compared to that of humans. Head and neck cancers in dogs and humans are relatively common and are often critical. Although the types of tumors developed frequently differ, the resulting cancer is still common. In dogs, it accounts for 7.2% of the tumors that occur. In humans, it was the seventh most common cancer globally in 2018. In the United States, it constitutes 3 and 1.5% of all cancer cases and deaths, respectively (22–24).

In human medicine, treatment of the head and neck cancer involves a surgical approach, RT and chemotherapy. These are performed either alone or in various combinations. Depending on the stage of the disease, anatomical site, or surgical accessibility, different treatments are chosen to ensure the optimal outcome and survival rate. In most cancer cases, RT is an essential option (23–27). In veterinary medicine, RT is also indicated in cancers where surgical access is difficult, with head and neck cancer accounting for a large proportion. Therefore, there are also some previous RT studies in veterinary medicine. However, unlike these previous studies, this study focuses on segmentation, the prerequisite process of RT (22, 28–31). This is because studies of automatic segmentation in dogs, particularly DLBAS, are insufficient (10–13, 15, 16).

The study developed an auto-segmentation tool using deep learning and evaluated the feasibility of the DLBAS method used for delineating RT planning for head and neck organs in dogs.

## Materials and Methods

### CT Image

The study was performed on the head and neck organs of 90 dogs referred to the Veterinary Medical Teaching Hospital, Konkuk University, from August 2015 to January 2021. The computed tomography (CT) data of 80 dogs were collected using a 4-channel helical CT scanner (LightSpeed®, GE Healthcare, Milwaukee, Wisc., USA). The CT data of 10 dogs were transmitted from other animal hospitals; the data were collected using 16-channel helical CT scanners. Post-contrast CT data were selected for this study. Dogs were positioned in sternal recumbency. Images were obtained with controlled respiration to minimize the artifacts caused by breathing. The acquisition parameters were as follows, depending on the size of the dog: kVp, 120; mA, 100–300; slice thickness and interval, 1.25–2.5 mm.

Classifications included are ages, body weight, skull patterns, cephalic index, and the presence of lesions in head and neck organs. Skull patterns of dogs were further divided into three categories: brachycephalic, mesocephalic, and dolichocephalic. The cephalic index was added as a criterion for a more objective evaluation.

**Figure 1:**
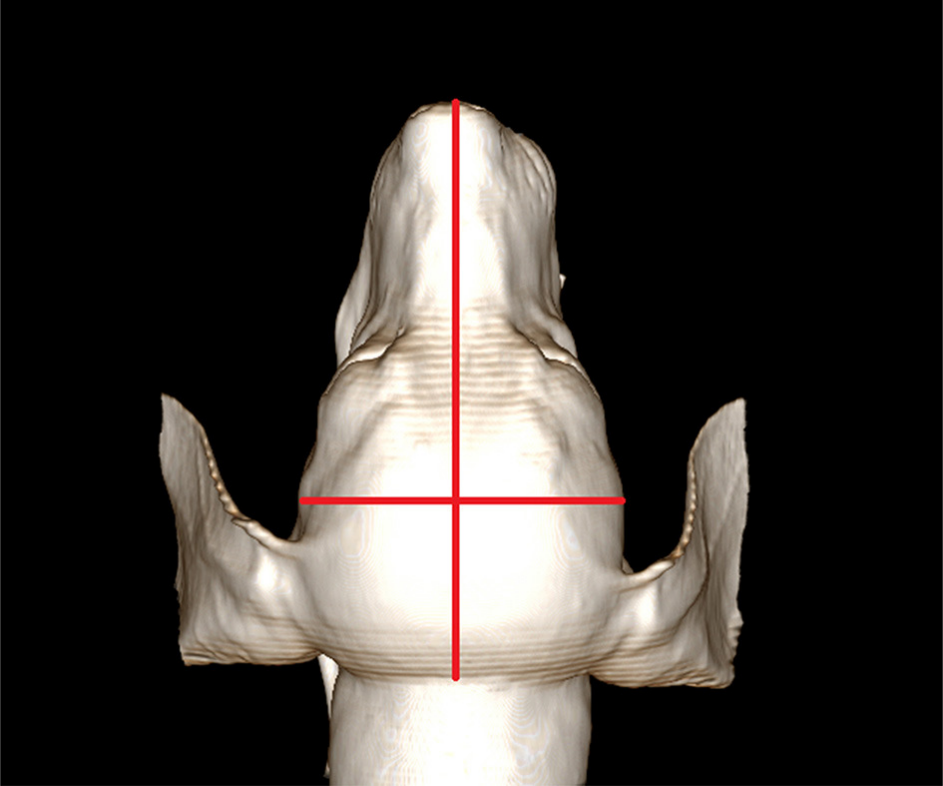
Measurement of the cephalic index. For measuring the cephalic index, skull width is measured between the left and right zygomatic arch. The skull length is measured from the nose tip to the occipital protuberance. The cephalic index is calculated as skull width/skull length. Here, the cephalic index of this dog is 0.57.

Head width and length were measured to calculate the cephalic index; cephalic index = head width/head length (Figure 1). All cephalic index values were measured using the reconstructed image of the head based on CT data.

The segmentation list for this study was prepared by considering potential organs at risk (OARs) in the heads and necks of dogs. It included various types of OARs: the eyes, lens, cochlea, temporomandibular joint, mandibular salivary gland, parotid salivary gland, pharynx and larynx, brain, and spinal cord. The region of interest (ROI) of this study was the second cervical vertebral level.

### Deep Learning-Based Automatic Segmentation

In this study, CT data from a total of 90 dogs were used. To develop the DLBAS algorithm, data from 80 dogs were included, 60 as training and validation sets and 20 as test sets. In addition, 10 clinical test sets were included for the evaluation of clinical feasibility. The expert contours used as ground truth for the 90 dogs were manually delineated by a single radiologist who has completed a master course in veterinary medical imaging. Radiologist worked as a radiologist for 2 years. For the 10 clinical test sets, two radiologists were added for the study. One of the radiologists completed a doctoral course in veterinary medical imaging and worked as a radiologist for 4 years and teaches veterinary anatomy at Konkuk University in Korea. Another radiologist is in the doctoral course of veterinary medical imaging and completed a master's course in veterinary surgery. This radiologist worked as a surgeon and radiologist for 4 and 2 years, respectively.

To ensure a robust network, the network fully matched the resolution of the CT image and adjusted the Hounsfield unit ([−100, 700] to [−1.0, 1.0]). The CT image was normalized to 1.0 × 1.0 × 3.0 mm^3^.

A two-step, three-dimensional (3D) fully convolutional DenseNet was developed to automatically contour the target structures, as originally proposed by Jegou et al. (32). The fully convolutional DenseNet network was trained on a computer equipped with a graphic processing unit (NVIDIA TITAN RTX GPU) with Tensor-flow 2.4.1 in Python 3.6.8. The two-step segmentation is namely localization and ROI segmentation. In the first step, each OAR was cropped concurrently through multilabel segmentation around each ROI in the preprocessed images. The localization model is preformed automatically. In the localization process, x, y, z directions were downsampled to half the reduction of image resolution. In the second step, each label segmentation was used for OAR from the first step. To minimize the margin of outside volume, all the x, y, z sizes were calculated, and each ROI segmentation volume was cut off. In the end, single-label segmentation was trained with the ROIs.

**Figure 2:**
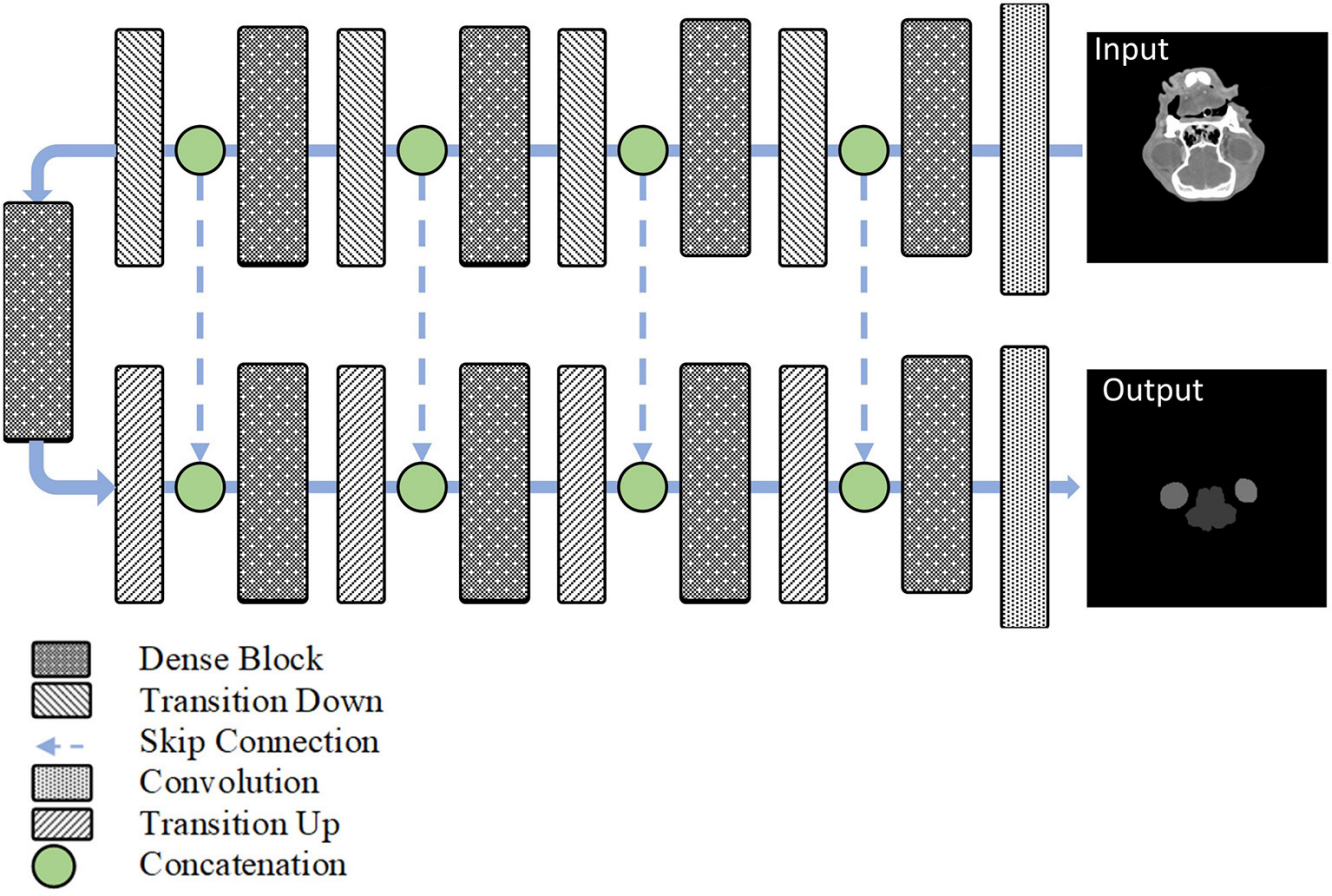
The architecture of the proposed fully convolutional DenseNet.

The fully convolutional DenseNet architecture consists of dense blocks similar to the residual blocks in a U-Net architecture (Figure 2). Following the convolution layer, the transition down layers consists of batch normalization, rectified linear units, 1 × 1 convolution, dropout (*p* = 0.2), and a 2 × 2 max-pooling operation. The skip connection components represent the concatenation of the feature maps from the downsampling path with those in the upsampling path, thereby ensuring a high-resolution output. Finally, the transition up layers consists of 3 × 3 deconvolutions with a stride of two to progressively recover the spatial resolution.

### Comparison Metrics

To test the accuracy of each segmentation model, 20 test sets and 10 clinical test sets were assessed with the Dice similarity coefficient (DSC) and the 95% Hausdorff distance (HD). A single radiologist delineated the manual contours; these were used as ground truths. The DSC metric quantifies the closeness of the automated and expert contours by dividing double the overlap of the two contours by the sum of their volumes (33), as follows:

$$\begin{array}{l} DSC=\;\frac{2\left(A\cap B\right)}{\left|A|\;+\;|B\right|} \end{array}$$

The range of DSC is [0,1]. A DSC of zero indicates no spatial overlap between two contours while one indicates an impeccable match. In this study, a minimal DSC of 0.75 was considered an acceptable match.

$$\begin{array}{l} H\left(A,B\right)=\max\left\{h\left(A,B\right),\;h\left(B,A\right)\right\} \end{array}$$

The surface distance of two contours at metric space is measured by the HD by calculating the maximum distance between a point in one contour and the closest point in the other contour.

The calculation of the 95th percentile of the distances between one contour and the other contour is denoted as HD95 (34).

### Evaluation of Clinical Feasibility

The DLBAS was trained on ground truth from annotator one. The proposed DLBAS was also evaluated for availability in cancer patients. The 10 clinical test sets were formed with a relatively large displacement of segmentations with mass or inflammation for verification in cancer patients. These clinical test sets were used to verify the network by comparing the results of DLBAS with the ground truth.

The proposed DLBAS was assessed by using comparison metrics, these were the DSC and HD metrics. The mean values and standard deviations (SD) were recorded for evaluation.

The clinical test sets were delineated by three radiologists as human annotators. Annotator one delineated segmentations manually; these were used as ground truth for the evaluation. In addition, segmentations delineated by the other annotators were assessed as HAs.

Three methods were included for this evaluation, the DLBAS predictions, the two HAs, and the two HAs with additional readjustments to the DLBAS predictions (HA_DLBASs). The DLBAS predicted the segmentations of 10 clinical test sets based on the ground truth. The HA_DLBASs were conducted by two annotators based on the predicted data of DLBAS. The two annotators only readjusted data that the DLBAS predicted inaccurately.

For analysis, DLBAS predictions, two HAs, and two HA_DLBASs were evaluated with comparison metrics. Comparison metrics included the DSC, HD, and contouring time. The accuracy and consistency were evaluated with mean values and SD, respectively.

The production times of DLBAS, HAs, and HA_DLBASs were recorded for the overall 15 OARs for efficacy evaluation. The production time of each method was measured in a different process.

1. DLBAS: Only the time for running each OAR was recorded; the time spent for pre-processing and training was excluded.
2. HAs: The time recorded started at the beginning of the contouring and finished at the end of the CT image.
3. HA_DLBASs: Based on the DLBAS predicted contours, we recorded the time spent to correct the contours.

## Results

This study included two variables, depending on the skull shape, cephalic index, and skull patterns. For the skull pattern, more than half of the dogs (59) had mesocephalic skulls, while 17 and 14 dogs had brachycephalic and dolichocephalic skulls, respectively. The cephalic index of 90 dogs measured ranged from 0.46 to 0.91, with an average value of 0.6. According to the cephalic index, data were divided into four ranges, with intervals of 0.1. The modal range (35) was 0.6–0.7 (Table 1).

**Table 1:** Distribution of numbers and proportions according to variables.

**Variables**	**Number (%)**			
	**Total**	**Training &** ** validation set**	**Test set**	**Clinical** ** test set**
	**90**	**60**	**20**	**10**
**Age (years)**	
0 ~ 3	14 (15.6)	10 (16.7)	3 (15.0)	1 (10.0)
3 ~ 6	10 (11.1)	8 (13.3)	1 (5.0)	1 (10.0)
6 ~ 10	45 (50.0)	30 (50.0)	11 (55.0)	4 (40.0)
10 ~	21 (23.3)	12 (20.0)	5 (25.0)	4 (40.0)
**Weight (kg)**	
1 ~ 10	71 (78.9)	47 (78.3)	15 (75.0)	9 (90.0)
10 ~ 20	11 (12.3)	7 (11.7)	4 (20.0)	-
20 ~ 30	6 (6.6)	4 (6.7)	1 (5.0)	1 (10.0)
30 ~	2 (2.2)	2 (3.3)	-	-
**Cephalic index**	
**(W/L)**	
0.4 ~ 0.5	4 (2.2)	2 (3.3)	2(10.0)	-
0.5 ~ 0.6	31 (36.7)	24 (40.0)	3 (15.0)	4 (40.0)
0.6 ~ 0.7	35 (38.9)	27 (45.0)	5 (25.0)	3 (30.0)
0.7 ~	20 (22.2)	7 (11.7)	10 (50.0)	3 (30.0)
**Skull pattern**	
Mesocephalic	59 (65.6)	41 (68.3)	11 (55.0)	7 (70.0)
Brachycephalic	17 (18.9)	6 (10.0)	9 (45.0)	2 (20.0)
Dolichocephalic	14 (15.5)	13 (21.6)	-	1 (10.0)
**Presence of lesions**	
Absence	63 (80.0)	54 (95.0)	9 (45.0)	0 (0.0)
Presence	27^a^ (20.0)	6^b^ (5.0)	11^c^ (55.0)	10^d^ (100.0)

*W/L, skull width/skull length;*

a *oral tumor, n = 7; nasal tumor, n = 6; otitis, n = 4; cervical tumor, n = 2, ear canal tumor, n = 2; mandibular tumor, n = 1; maxillary tumor, n = 1; orbital tumor, n = 1; sialadenitis, n = 1; sialocele, n = 1; zygomatic none tumor, n = 1;*

b *oral tumor, n = 3; otitis, n = 2; ear canal tumor, n = 1;*

c *oral tumor, n = 4; cervical tumor, n = 1; maxillary tumor, n = 1; nasal tumor, n = 1; orbital tumor, n = 1; otitis, n = 1; sialadenitis, n = 1; zygomatic bone tumor, n = 1;*

d *cervical tumor, n = 1; ear canal tumor, n = 1; mandibular tumor, n = 1; nasal tumor, n = 5, otitis, n = 1; sialocele, n = 1*.

Table 2 showed that most of the relations of the variables had no difference compared to mean DSC and mean HD (Table 2). The average DSC and HD values were 0.83 ± 0.01 and 2.71 ± 0.31 mm, respectively. All the age ranges had the same DSC of 0.83. It also showed approximate results for a mean HD of 2.71. Most of the other variables, such as weight, skull pattern, and presence of lesion also showed no significant difference from the average. On the other hand, the cephalic index was significantly different (0.21) from the mean DSC (0.62) for the range 0.5–0.6. Furthermore, the mean HD also showed a significant difference (0.72).

**Table 2:** Accuracy correlation according to variables in the test set.

**Variables**	**Score (mean** **±** **SD)**	
	**DSC**	**HD (mm)**
	**0.83 ± 0.01**	**2.71 ± 0.31**
Age (years)
0 ~ 3	0.83	2.91
3 ~ 6	0.83	2.75
6 ~ 10	0.83	2.68
10 ~	0.83	2.63
Weight (kg)
1 ~ 10	0.83	2.75
10 ~ 20	0.84	2.56
20 ~ 30	0.82	2.61
30 ~	-	-
Cephalic index (W/L)
0.4 ~ 0.5	0.83	2.44
0.5 ~ 0.6	0.62	1.99
0.6 ~ 0.7	0.84	2.80
0.7 ~	0.75	2.48
Skull pattern
Mesocephalic	0.84	2.69
Brachycephalic	0.82	2.73
Dolichocephalic	-	-
Lesion
Presence	0.83	2.76
Absence	0.83	2.67

*SD, standard deviation; DSC, Dice similarity coefficient; HD, Hausdorff distance; W/L, skull width/skull length*.

**Figure 3:**
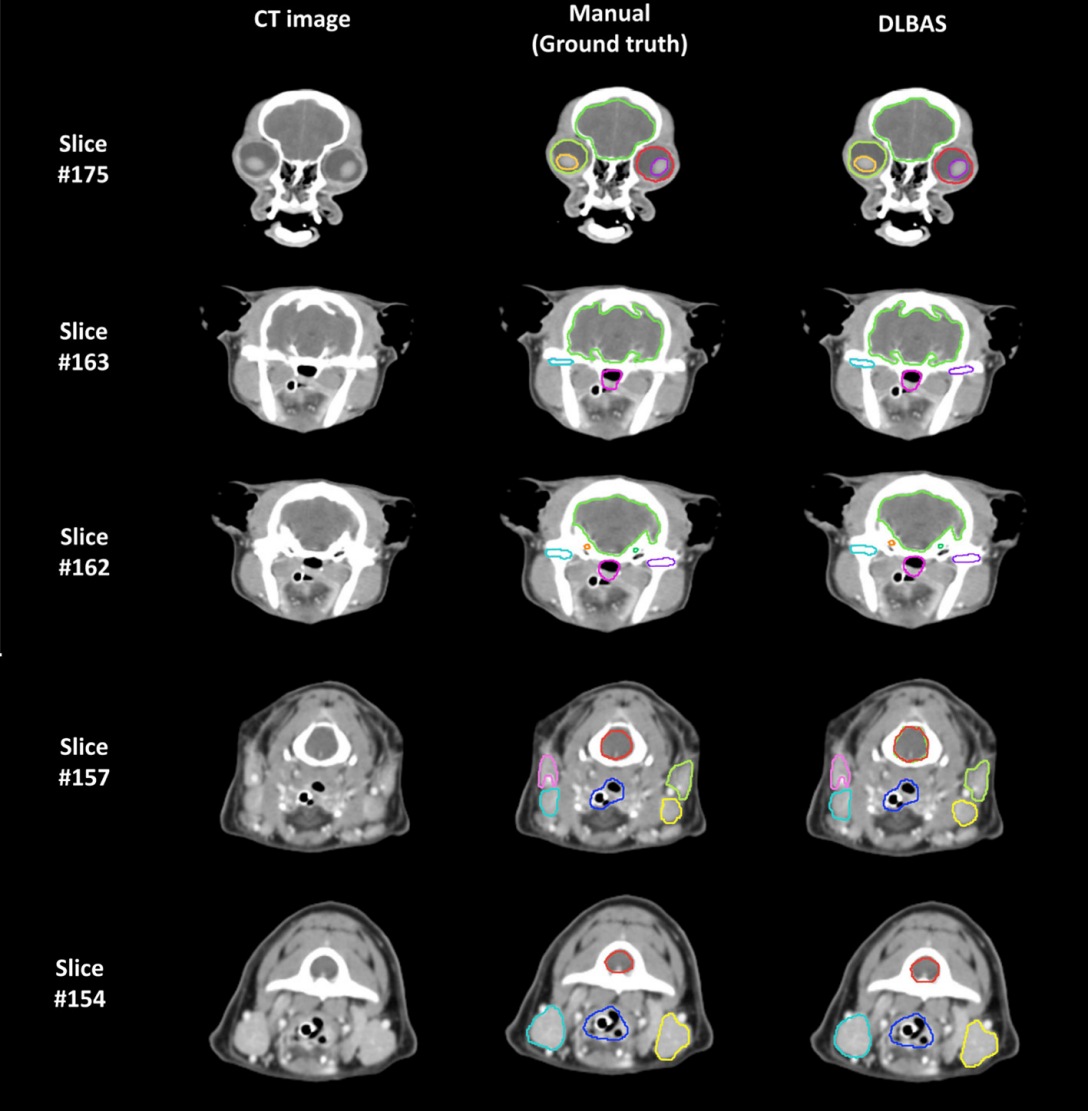
Examples of the ground truth and deep-learning-based automatic segmentation in a test set (DLBAS). Segmentations can be identified in each slice. For the DLBAS, it is difficult to identify a significant difference. Slice #175 shows the eye (red, lime green), lens (yellow, purple), and brain (yellow, green). Slice #163 and #162 show the brain (yellow, green), cochlear (orange, green), temporomandibular joint (sky blue, purple), and pharynx and larynx (pink). Slice #157 and #154 show the mandibular salivary gland (sky blue, yellow), parotid salivary gland (pink, lime green), pharynx and larynx (blue), and spinal cord (red). There are visible differences between the temporomandibular joint (purple) in Slice #163 and the spinal cord (red) in slice #157. Especially, the predicted DLBAS spinal cord (red) region in slice #157 overlapped with the brain (green).

**Figure 4:**
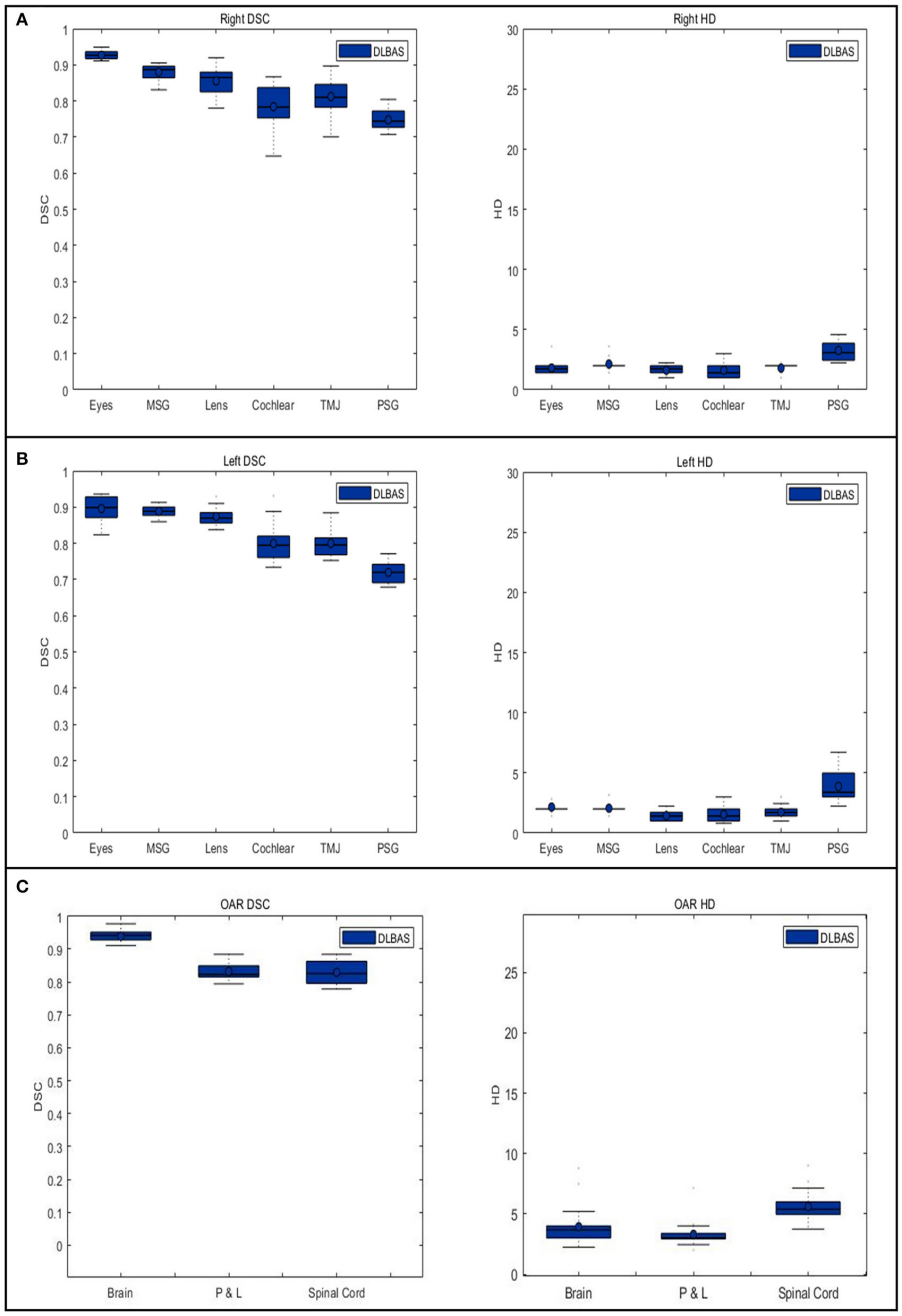
Boxplots of the Dice similarity coefficient and Hausdorff distance in each organ at risk are obtained from deep-learning-based automatic segmentation. **(A)** right organs, **(B)** left organs, and **(C)** other OARs. DSC, Dice similarity coefficient; HD, Hausdorff distance; OAR, organ at risk; MSG, mandibular salivary gland; TMJ, temporomandibular joint; PSG, parotid salivary gland; P & L, pharynx, and larynx.

The right eye among 15 OARs showed the highest accuracy. The mean DSC was 0.93 and the mean HD was 1.80. The lowest accuracy was recorded for the left parotid salivary gland, with 0.72 and 3.88. The DLBAS model showed reliable DSC, HD values, and also a short contouring time of ~3 s for all OARs. The performance of the DLBAS is shown in many slices (Figure 3). The average DSC, HD, and SD about each OAR are displayed in the boxplots (Figure 4). The average DSC and HD values were 0.83 ± 0.01 and 2.71 ± 0.31 mm, respectively.

In this study, except for the right cochlear and bilateral parotid salivary gland, all OARs exceeded the DSC value of 0.79. In addition to the bilateral parotid salivary gland, three OARs, the brain, pharynx and larynx, and spinal cord showed an inaccurate HD value of 3.18.

Using the proposed DLBAS, DSC and HD values were obtained for all clinical test sets (Tables 3, 4). All variables were calculated using the manual contours of HA one as the ground truth. The DLBAS of the clinical test sets showed lower DSC (0.78 ± 0.11) and higher HD (4.30 ± 3.30 mm) values compared to the test sets. The lowest accuracy recorded among the OARs for the DSC and HD was right cochlear (0.50 ± 0.28) and left parotid salivary gland (7.01 ± 8.67 mm), respectively. The highest accuracy recorded for the DSC and HD was the brain (0.90 ± 0.11) and the right eye (2.00 ± 0.71 mm), respectively.

**Table 3:** Dice similarity coefficient of each clinical test set obtained from deep-learning-based automatic segmentation.

**OAR**	**Group 1**				**Group 2**					
	**C1**	**C2**	**C3**	**C4**	**C5**	**C6**	**C7**	**C8**	**C9**	**C10**
Lens (L)	0.64	0.70	0.70	0.89	0.89	0.89	0.89	0.89	0.90	0.87
Lens (R)	0.90	0.93	0.93	0.66	0.86	0.87	0.86	0.78	0.88	0.85
Eye (L)	0.85	0.90	0.90	0.64	0.94	0.95	0.94	0.92	0.95	0.93
Eye (R)	0.95	0.24	0.24	0.55	0.95	0.94	0.93	0.93	0.95	0.94
Cochlear (L)	0.68	0.41	0.41	0.64	0.64	0.63	0.47	0.71	0.60	0.87
Cochlear (R)	0.65	0.05	0.05	0.27	0.57	0.65	0.65	0.71	0.59	0.84
TMJ (L)	0.52	0.90	0.70	0.90	0.92	0.90	0.85	0.86	0.89	0.88
TMJ (R)	0.41	0.31	0.31	0.61	0.87	0.87	0.87	0.71	0.88	0.86
MSG (L)	0.68	0.55	0.55	0.50	0.90	0.93	0.93	0.73	0.93	0.85
MSG (R)	0.74	0.82	0.82	0.90	0.90	0.92	0.95	0.72	0.94	0.82
PSG (L)	0.35	0.84	0.84	0.85	0.85	0.91	0.83	0.86	0.89	0.81
PSG (L)	0.48	0.68	0.68	0.43	0.43	0.90	0.82	0.91	0.88	0.78
Pharynx& larynx	0.83	0.96	0.77	0.77	0.94	0.95	0.95	0.89	0.95	0.84
Brain	0.63	0.77	0.89	0.90	0.97	0.97	0.97	0.94	0.97	0.95
Spinal cord	0.74	0.89	0.71	0.71	0.90	0.90	0.88	0.81	0.89	0.89
**Total**	0.67	0.66	0.63	0.68	0.83	0.88	**0.85**	0.83	0.87	0.87

*OAR, organ at risk; TMJ, temporomandibular joint; MSG, mandibular salivary gland; PSG, parotid salivary gland; L, left; R, right; C1–C10, clinical test set 1–10*.

**Table 4:** Hausdorff distance of each clinical test set obtained from deep-learning-based automatic segmentation.

**OAR**	**Group 1**				**Group 2**					
	**C1**	**C2**	**C3**	**C4**	**C5**	**C6**	**C7**	**C8**	**C9**	**C10**
Lens (L)	0.81	39.53	1.67	3.69	2.69	1.69	1.89	0.69	0.83	1.89
Lens (R)	0.81	1.73	7.70	2.09	2.69	1.69	1.86	0.69	0.83	1.86
Eye (L)	0.81	2.52	3.38	9.26	3.09	1.69	1.94	1.43	0.83	1.94
Eye (R)	0.81	1.97	2.89	2.09	3.35	1.69	1.95	1.93	1.38	1.93
Cochlear (L)	1.15	2.08	3.32	2.09	2.69	1.69	1.64	0.99	2.66	1.47
Cochlear (R)	0.81	6.27	2.49	2.09	2.69	1.69	1.57	0.69	0.83	1.65
TMJ (L)	5.01	1.34	10.13	5.37	2.69	1.69	1.92	0.69	0.83	1.85
TMJ (R)	2.53	20.28	2.83	5.86	2.69	1.69	1.87	0.69	0.83	1.87
MSG (L)	15.10	7.11	9.47	7.46	2.69	4.77	1.90	0.69	3.71	1.93
MSG (R)	12.29	3.76	6.67	8.14	2.69	4.37	1.90	0.69	1.38	1.95
PSG (L)	11.90	5.29	5.82	29.91	2.98	1.69	1.85	1.94	6.86	1.83
PSG (L)	8.49	8.15	4.44	17.95	2.99	10.59	1.43	3.72	2.72	1.82
Pharynx& larynx	10.00	3.93	9.83	8.51	3.29	1.99	1.94	2.74	0.83	1.95
Brain	8.53	4.05	8.09	30.49	6.20	2.00	1.97	1.76	0.83	1.97
Spinal cord	1.28	4.20	8.79	38.90	2.98	1.69	1.90	0.69	1.17	1.88
**Total**	5.36	7.48	5.83	11.59	3.09	2.71	1.83	1.34	1.77	1.85

*OAR, organ at risk; TMJ, temporomandibular joint; MSG, mandibular salivary gland; PSG, parotid salivary gland; L, left; R, right; C1–C10, clinical test set 1–10*.

The results were split into two groups. Group 1 showed low accuracy, while group 2 showed high accuracy. Group 1 included four out of the ten clinical test sets, while the other six were included in group 2. Group 1 showed an average DSC of 0.66 and an average HD of 7.57. Group 2 scored 0.86 and 2.10 for the DSC and HD, respectively. Comparing the two groups, the difference of the DSC is 0.2 while for the HD it is 5.47.

**Figure 5:**
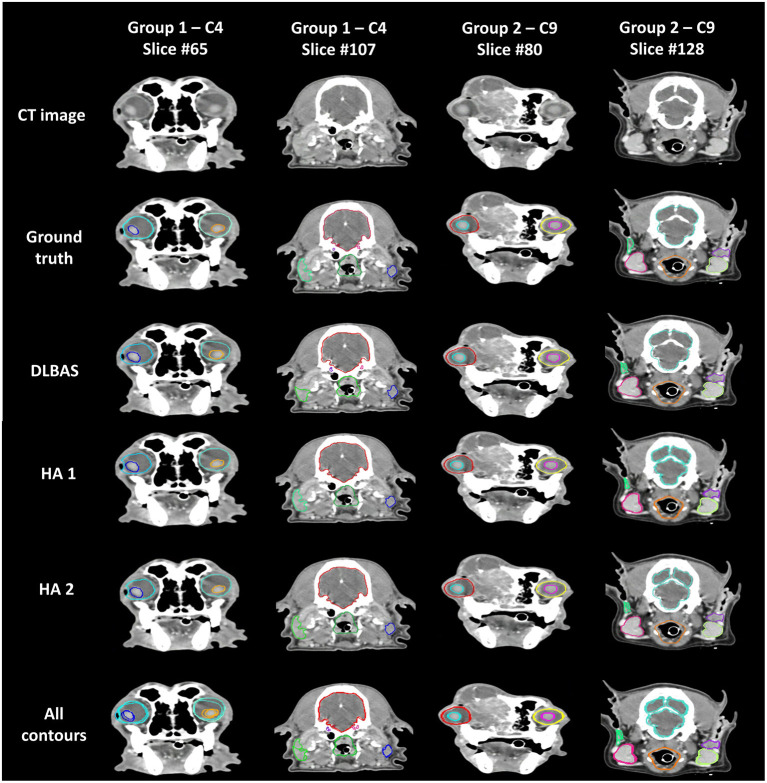
Examples of ground truth deep-learning-based automatic segmentation, and human annotations used in clinical test sets in groups 1 and 2. All contours of the three methods are combined and displayed on each slice. Slice #65 shows the eye (aqua, aquamarine), and lens (blue, orange). Slice #107 shows the brain (red), cochlear (purple, pink), parotid salivary gland (lime, blue), and pharynx and larynx (green). Slice #80 shows the eye (red, yellow), and lens (aqua, pink). Slice #128 shows the brain (aquamarine), mandibular salivary gland (red, yellow-green), parotid salivary gland (green, purple), and pharynx and larynx (orange). CT, computed tomography; DLBAS, deep learning-based automatic segmentation; HA, human annotation.

The difference between ground truth, DLBAS, and the HAs in groups 1 and 2 are shown in Figure 5. For the DLBAS of group 1, most of the predicted contours were in a different position, compared to those of group 2. Furthermore, in group 1, the positions of OARs changed owing to cancer and inflammation. However, for group 2, most of the organs remained in their original positions. The difference between the ground truth and HAs was difficult to ascertain, however differed to the predictions DLBAS. In addition, the difference between the two HAs was insignificant. When all the contours are combined, group 1 is identified by multiple lines, unlike group 2.

The overall results of HA, DLBAS, HA_DLBAS are summarized in Tables 5, 6. The results were obtained by comparing results to the ground truth. The HA_DLBAS presented the most reliable DSC and HD values (DSC: 0.94 ± 0.04 and HD: 2.3 ± 0.56 mm). Next were HA (DSC: 0.85 ± 0.07 and HD: 2.74 ± 1.11 mm) and DLBAS (DSC: 0.78 ± 0.11 and HD: 4.29 ± 3.30 mm).

**Table 5:** Dice similarity coefficient by three contouring methods of the clinical test set.

**OAR**	**DSC (mean** **±** **SD)**		
	**HA**	**DLBAS**	**HA_DLBAS**
Lens (L)	0.85 ± 0.04	0.83 ± 0.10	0.87 ± 0.04
Lens (R)	0.85 ± 0.07	0.85 ± 0.08	0.93 ± 0.06
Eye (L)	0.93 ± 0.09	0.89 ± 0.09	0.92 ± 0.02
Eye (R)	0.93 ± 0.07	0.76 ± 0.30	0.95 ± 0.09
Cochlear (L)	0.81 ± 0.08	0.61 ± 0.14	0.92 ± 0.06
Cochlear (R)	0.73 ± 0.18	0.50 ± 0.28	0.94 ± 0.03
TMJ (L)	0.77 ± 0.15	0.83 ± 0.13	0.88 ± 0.08
TMJ (R)	0.80 ± 0.11	0.67 ± 0.24	0.81 ± 0.07
MSG (L)	0.89 ± 0.04	0.76 ± 0.18	0.98 ± 0.02
MSG (R)	0.89 ± 0.05	0.85 ± 0.08	0.99 ± 0.03
PSG (L)	0.83 ± 0.05	0.80 ± 0.16	0.97 ± 0.03
PSG (R)	0.79 ± 0.19	0.70 ± 0.19	0.95 ± 0.04
Pharynx& larynx	0.87 ± 0.04	0.89 ± 0.08	0.99 ± 0.01
Brain	0.97 ± 0.09	0.90 ± 0.11	0.99 ± 0.02
Spinal cord	0.88 ± 0.07	0.83 ± 0.08	0.97 ± 0.02
**Total**	**0.85** **±** **0.07**	**0.78** **±** **0.11**	**0.94** **±** **0.04**

*OAR, organ at risk; HA, human annotation; DLBAS, deep-learning-based automatic segmentation; HA_DLBAS, human annotation with additional readjustments to DLBAS predictions; SD, standard deviation; TMJ, temporomandibular joint; MSG, mandibular salivary gland; PSG, parotid salivary gland; L, left; R, right; C1–C10, clinical test set 1–10*.

**Table 6:** Hausdorff distance by three contouring methods of the clinical test set.

**OAR**	**HD (mean** **±** **SD, mm)**		
	**HA**	**DLBAS**	**HA_DLBAS**
Lens (L)	2.94 ± 3.47	5.54 ± 11.98	1.95 ± 0.52
Lens (R)	1.94 ± 0.22	2.20 ± 2.04	1.90 ± 1.48
Eye (L)	1.73 ± 0.46	2.69 ± 2.46	2.79 ± 0.51
Eye (R)	1.71 ± 1.04	2.00 ± 0.71	2.30 ± 0.35
Cochlear (L)	1.44 ± 0.77	1.98 ± 0.74	1.61 ± 0.30
Cochlear (R)	1.41 ± 2.14	2.08 ± 1.63	2.31 ± 0.69
TMJ (L)	2.80 ± 1.76	3.15 ± 2.93	2.40 ± 0.53
TMJ (R)	2.10 ± 1.91	4.11 ± 5.86	2.43 ± 0.42
MSG (L)	2.63 ± 2.07	5.48 ± 4.41	1.38 ± 1.12
MSG (R)	3.30 ± 1.03	4.38 ± 3.65	2.18 ± 0.43
PSG (L)	3.32 ± 2.01	7.01 ± 8.67	2.11 ± 0.03
PSG (R)	4.82 ± 2.27	6.23 ± 5.16	2.62 ± 0.04
Pharynx& larynx	4.90 ± 0.57	4.50 ± 3.53	3.30 ± 0.46
Brain	3.32 ± 0.86	6.59 ± 8.84	3.54 ± 1.24
Spinal cord	2.72 ± 1.15	6.35 ± 11.98	1.72 ± 0.22
**Total**	**2.74** **±** **1.11**	**4.29** **±** **3.30**	**2.30** **±** **0.56**

*OAR, organ at risk; HA, human annotation; DLBAS, deep-learning-based automatic segmentation; HA_DLBAS, human annotation with additional readjustments to DLBAS predictions; SD, standard deviation; TMJ, temporomandibular joint; MSG, mandibular salivary gland; PSG, parotid salivary gland; L, left; R, right; C1–C10, clinical test set 1–10*.

There was a significant time reduction when comparing DLBAS to the HAs, HA_DLBAS for contouring of 15 OARs (Table 7). The average time spent for HA, DLBAS, and HA_DLBAS was 80, 0.05, and 30 min, respectively. Using DLBAS, the contouring time was expected to be reduced 1,800 times. Using HA_DLBAS, the highest DSC and the lowest HD values were recorded, and the contouring time was reduced by more than half. For the HA_DLBAS procedure, most of the predicted images of DLBAS needed a short time to readjust the segmentation. However, those in group 1 segmentation needed at least five times more time than those in group 2.

**Table 7:** Comparison of contouring times.

**Contouring method**	**Contouring time (min)**
DLBAS	0.05 ± 0.01
HA	80 ± 25.00
HA_DLBAS	30 ± 12.28

*DLBAS, deep-learning-based automatic segmentation; HA, human annotation; human annotation with additional readjustments to DLBAS predictions*.

## Discussion

Medical image processing technology based on artificial intelligence has evolved from simple image detection technology to advanced automatic image processing technology. These technologies are advantageous as they can reduce the workload and save time for tasks that require human intervention. In particular, the manual delineation for segmentation of anatomical structures in RT planning procedure is not only a tedious task, but also inherently difficult for experts (7). Although not for RT planning, automatic segmentation methods have been evaluated, including atlas-based automatic segmentation and triple cascaded convolutional neural networks for mice and rats (7, 35). Incorporating a more advanced form of DLBAS into RT planning has not yet been applied to veterinary medicine. This study is the first to apply methods based on deep learning technology to RT planning in dogs. Furthermore, the results of this study confirm that automatic segmentation can be achieved with high accuracy and a short contouring time.

To avoid unnecessary irradiation to critical anatomical structures and OARs, establishing an accurate segmentation is an important factor in RT planning. However, considering individual differences or the various head shapes and sizes of dogs, it can be sufficiently predicted that the segmentation accuracy will be affected (36). Thus, in the process of setting up 80 training and validation sets, various skull shapes were included, and it was predicted to have been learned accurately during the deep learning process. In this study, the results of DLBAS showed reliable accuracy regardless of differences in skull shapes. Although the accuracy was relatively low when the cephalic index range was 0.5–0.6, there was no significant difference. In addition, it was found that age, weight, and the presence of lesions did not affect the deep learning results.

The DLBAS proved to be robust and reliable in automatic segmentation as the results were very similar to the ground truth. The mean DSC and HD values of this study are similar to those recorded in previous human studies (DSC = 0.79 and HD = 3.18 mm) (31). In the case of OARs with high accuracy, the boundaries were distinctly common and the variation among the test sets was small. In particular, the brain was surrounded by skulls with distinct differences in contrast, and this allowed accurate predictions of the segmentation. In contrast, OARs with low accuracy were in small volume and varied across the different shapes among the test sets. The cochlear was present in up to three slices on the CT images, therefore, it was difficult to distinguish its exact location in all segmentation methods in this study. Furthermore, the parotid salivary glands were the most diverse in shape, and thus reduced the consistency in the training process of deep learning. This study further goes on to support that the DLBAS methods used in human medicine are likely more accurate and faster than the atlas-based automatic segmentation method (3). Therefore, even in dogs, DLBAS is superior to other automatic segmentation methods including atlas-based automatic segmentation.

The DLBAS method was applied to tumor patients in test sets, resulting in a successful automatic segmentation. Therefore, the DLBAS method confirmed that there was no significant difference in the accuracy of automatic segmentation with or without tumors. However, the mean DSC value decreased significantly in the three clinical sets whose cephalic index values ranged from 0.5 to 0.6. As a result of checking the CT image of clinical sets, it can be determined that the displacement or deformity of the anatomical structure is more likely owing to the tumor lesion than the cephalic index. Therefore, further evaluations were needed to determine whether the application of DLBAS was possible if the displacement and deformation of the organs due to lesions were severe.

Despite the presence of displacements and deformations of organs in the clinical test set, DLBAS was identified as a reliable segmentation method and showed similar accuracy to ground truth. However, the accuracy decreased significantly in group 1 owing to two main reasons. First, unclear segmentation, such as when the surroundings respond to inflammation and tumors, or when contrast enhancement was insufficient. For example, insufficient contrast enhancement intensity of the salivary gland, which is usually lower than the average HU value, can affect the accuracy of the segmentation. Second, the left and right asymmetry of the CT scan. This is because of the displacement of OARs or inaccurate CT scan posture by large lesions. Thus, this resulted in inaccurate localization during the two-step segmentation process, leading to reduced accuracy. Failure to localize one or more OARs also led to lower accuracy. However, despite these conditions, DLBAS has proven to be remarkably accurate in its evaluation of clinical feasibility. Therefore, the DLBAS tool proposed herein is capable of high accuracy in automatic segmentation while also completing the segmentation quickly with minimal intervention from experts.

There is a process to evaluate the additional clinical feasibility of DLBAS with expert interventions. The HA_DLBAS method showed higher accuracy and consistency compared to that of DLBAS and HAs. In addition, a comparison of contouring times shows that HA_DLBAS takes less time than the HAs. A previous study shows that the results of segmentation from multiple observers overlapped with up to 60% volume variations that could lead to substantial differences in RT planning (9). Therefore, whether expert intervention can lead to higher accuracy and improve interobserver consistency was evaluated. This was confirmed by the better comparison metrics and small SD in the HA_DLBAS method. These results imply that DLBAS, as a supplementary tool, can also be highly efficient.

This study has several limitations. First, additional verification of pre-contrast CT data is required. A previous study has shown that using post-contrast CT data can achieve higher accuracy in both manual and automatic segmentation (7). For this reason, only post-contrast CT data were selected for this study. However, because insufficient contrast enhancement could have reduced accuracy, as shown in group 1, further studies are needed to demonstrate the effect of contrast. Second, the number of data used for this study was insufficient. More CT data of dogs were initially collected. However, a number of these data were found to be defective during the screening process and had to be excluded. In addition, cases showing complete loss of OARs due to lesions were excluded. Cases with prosthetic implants were excluded owing to CT contrast differences in the eyeball. Thirdly, there are head and neck organs that were not included in the segmentation. The incidence of head and neck cancers in dogs is relatively high in the oral cavity, skull, and nasal cavity, and should have been included in segmentation (22). However, this study excluded these segmentations because of software limitations that failed to set thresholds.

## Conclusion

In conclusion, this study shows that DLBAS is capable of automatic segmentation of organs present in the heads and necks of dogs and can be utilized as a useful RT segmentation tool. The proposed algorithm itself proved to be robust and provided reliable automatic segmentation results. Therefore, DLBAS has great potential as a single or supporting tool for key processes of RT planning, making it a useful tool for optimizing the clinical workload and reducing labor load.

## Data Availability Statement

The raw data supporting the conclusions of this article will be made available by the authors, without undue reservation.

## Ethics Statement

The animal study was reviewed and approved by Kidong Eom Konkuk university. Written informed consent was obtained from the owners for the participation of their animals in this study.

## Author Contributions

JP, BC, KE, and JSK conceived the study and performed all the analysis and drafted the manuscript. JKo, JayK, and JaeK critically reviewed the contours as an annotator and contributed to the clinical analysis. JC, IP, and JL critically reviewed the text and contributed to the clinical analysis. All authors contributed to the article and approved the submitted version.

## Conflict of Interest

The authors declare that the research was conducted in the absence of any commercial or financial relationships that could be construed as a potential conflict of interest.

## Publisher's Note

All claims expressed in this article are solely those of the authors and do not necessarily represent those of their affiliated organizations, or those of the publisher, the editors and the reviewers. Any product that may be evaluated in this article, or claim that may be made by its manufacturer, is not guaranteed or endorsed by the publisher.
